# How could we make nutrition in the intensive care unit simple?

**DOI:** 10.5935/0103-507X.20160070

**Published:** 2016

**Authors:** Pierre Singer, Jonathan Cohen

**Affiliations:** 1 General Intensive Care Department, Rabin Medical Center, Sackler School of Medicine, Tel Aviv University - Tel Aviv, Israel.

## Introduction

A uniform approach may be applied to any process which may be defined as *simple* i.e. one which is orderly, easily understood, repeatable and reproducible and not complicated or complex. The approach to nutrition for critically ill patients in the intensive care unit (ICU) cannot be described as uniform or simple for a number of reasons. These patients frequently present with multiple, simultaneous problems and their course may be dynamic, unordered, complex, coherent only in retrospect and not repeatable. In addition, their nutritional status may vary from normal to moderate or even severe malnourishment, be influenced by the presence of co-morbidities such as obesity, cancer, or the sarcopenia related to age and may vary over the ICU course in the presence of changing organ function.

In order to advance optimal nutritional support as an integral part of the treatment plan of critically ill ICU patients, what is required is a pragmatic approach which, while taking into account their complexity, provides a uniform, simple approach which can be readily applied. The aim of this article is to suggest such an approach^(1)^ with consideration given to screening and assessment, therapy and monitoring of nutritional support.

## Who are the patients requiring nutritional support?

**Figure 1:**
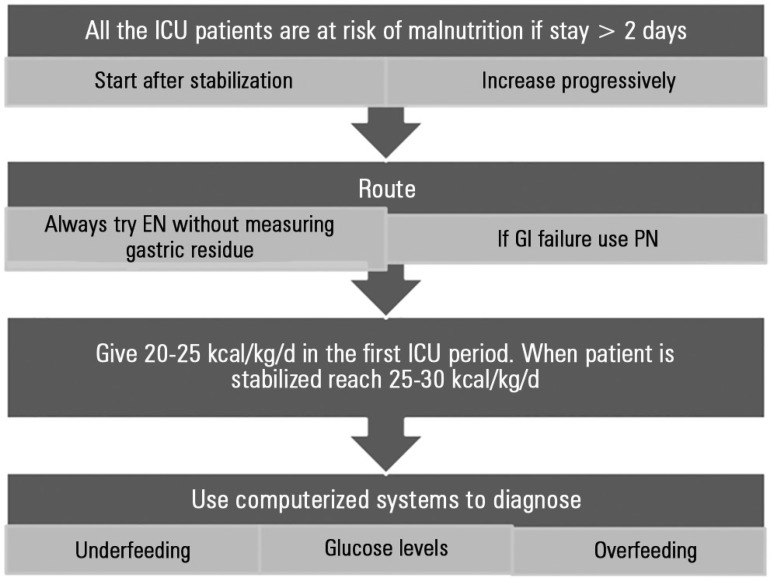
Flow chart to achieve nutritional support effectiveness in critically ill patients.

All ICU patients are defined as "at risk of malnutrition" according to the Nutritional Risk Screening 2002 - European Society for Clinical Nutrition and Metabolism (NRS 2002 - ESPEN) screening tool^(2)^ which takes into account body mass index (BMI), presence of weight loss and an acute disease which is always present in ICU patients. The subjective global assessment is useful to diagnose malnutrition and includes loss in weight, loss in muscle mass, in muscle function and fat loss. Simply put, all patients admitted to the ICU who have an anticipated stay of > 2 days require nutritional support. It is however mandatory to detect the patients with a BMI < 18.5kg/m^2^ or weight loss, regardless of BMI. These patients require a more aggressive approach (Figure 1).

EN - enteral nutrition; PN - parenteral nutrition; GI - gastrointestinal tract; ICU - intensive care unit.

## How much to prescribe?

According to the Nutrition Day ICU audit, there was no standard prescription in the 9,777 patients screened,^(3)^ suggesting that many patients are either over- or underfed. Underfeeding has a negative impact on clinical outcomes while overfeeding results in an increase in blood sugar, VCO_2_ production, length of ventilation as well as infections. Many predictive equations are available to plan energy requirements. However, their accuracy is low and this may translate to a both large positive or negative energy balance^(4)^ when compared to an assessment using indirect calorimetry. Since indirect calorimetry is not available in most ICUs, we recommend using a simple equation: 20 to 25kcal/kg/day and 1.2 to 2.0g/kg/day of protein.^(5)^ The actual weight should be used if the BMI is low while ideal weight is used when the BMI > 30.

To improve the detection of over or under nutrition, computerized monitoring of protein and energy delivery is very useful and is now more widely available. We have used this approach to show the association between negative energy balance and increased complications.^(6)^ A recent French study of ICU patients utilized a computerized system to support decision making regarding the achievement of nutrition goals.^(7)^ They showed that compared to historical controls, more patients in the computer-assisted group achieved 80% of the nutrition goals for both calories (79% *versus* 45%, p < 0.001) and nitrogen (37% *versus* 3%, p < 0.001). In addition, the incidence of nosocomial infections decreased from 59% to 41%, p < 0.03) in the computer-assisted group.

## When to start?

Nutritional therapy is not always started early, i.e. within the first 24 hours of ICU admission. The reason is that nutrition is not necessarily the first therapeutic priority of the treating physicians. Instead, nutritional support is generally started later in the ICU course, is slow to reach a designated target and practitioners are reluctant to prescribe parenteral nutrition where enteral nutrition does not meet the metabolic needs.

## Which route to choose?

Enteral feeding is the preferred route in critically ill patients who have no immediate contraindications for utilizing the gastrointestinal tract and as soon as respiratory and hemodynamic stabilization have been achieved. The administration of high doses of vasopressor does not appear to be a contraindication to enteral feeding as long as the patient displays signs of stability, as observed by Reigner et al. in > 3,032 patients with shock.^(8)^ In this study, eterally fed patients had improved survival at 29 days but an increase in the incidence of ventilator-associated pneumonia. The main contraindications for enteral feeding are hemodynamic instability, increasing or persistently elevated lactate levels (suggesting possible bowel ischemia), active gastrointestinal bleeding, ileus and severe diarrhea, abdominal compartment syndrome and short bowel syndrome. In these conditions, parenteral nutrition should be commenced.

As stated previously, the main pitfall of enteral nutrition is the lack of achieving nutritional targets. According to most surveys, only 50 to 60% of the target is achieved using the enteral route alone,^(9)^ leading to severe energy imbalance that may be associated with increased complications. For a long period, parenteral nutrition was considered as an evil. However, recently an elegant study comparing enteral to parenteral route in a large British multicenter study showed almost no differences in terms of complications between enteral and parenteral nutrition, encouraging the use of parenteral nutrition if enteral nutrition fails to achieve targets.^(10)^ The concept of supplemental parenteral nutrition is now accepted but the decision of when to start remains open to discussion: after 48 - 72 hours or after 7 to 10 days of failure of adequate enteral feeding. No well designed study has yet answered this question but many experts suggest starting early.^(11)^ The recent American Society for Parenteral and Enteral Nutrition (ASPEN) guidelines remain cautious in the face of a lack of clear evidence.^(12)^

## How to choose the nutrients?

There is no" one size fits all" formula for either enteral or parenteral nutrition.^(13)^ However, the highest priority should be the protein intake and therefore enteral formulas should be selected according to their protein content to reach the recommended amount: 1.2 to 2.0g/kg/day. Most formulae do NOT reach these levels and should be avoided. Of the remaining formulae, some use polymeric nutrients and are suitable for most cases. Others are composed of a semi-elemental diet and should be preferred in patients with malabsorption or long-term starvation. In specific cases requiring water restriction, such as acute renal or respiratory failure, formulae with higher caloric concentrations (1.5 to 2kcal/cc) can be used successfully.

Parenteral nutrition should also be prescribed according to amino acid content, giving preference to formulae delivering the highest protein content. Decreasing the carbohydrate content and using intravenous fat emulsions enriched in Ω-9 or Ω-3 polyunsaturated fatty acids is preferred in order to reduce the oxidative stress related to n-6 polyunsaturated fatty acids. These formulae appear to reduce the infection rate and length of stay of critically ill patients.^(5)^

## How to monitor?

Today, most ICUs are equipped with computerized information systems enabling the automatic monitoring and storage of vital signs, fluid and nutritional balances as well as quality indicators. Nutritional goals, which may be integrated into these systems, should include energy and protein intakes compared to the target; the amount of carbohydrates and lipids administered, including commonly administered non-nutritional calories, such as those derived from propofol, dextrose infusions and citrate administration during continuous renal replacement therapy. This will facilitate the early recognition of overfeeding and consequences of lipid and carbohydrate overloading. Many studies have demonstrated the usefulness of this approach.^(14)^ Glucose control remains an important and mandatory goal. Although the definitive glucose target has not yet been defined in the ICU, it is accepted that the serum glucose level should not exceed 180mg/dL while hypoglycemia should be strenuously prevented. In addition, large glycemic variability has been associated with increased mortality and should also be avoided.^(15)^ Recently, computerized systems have been proposed to support ICU decision- making regarding insulin administration and fine tuning. This has been shown to decrease the percentage of glucose levels > 180 and < 60mg/dL as well as decreasing glucose variability.^(16)^ In addition, the time to reach stability was decreased. Clearly the use of computerized systems may be of great help in the ICU setting where complexity is more and more frequent.

The monitoring of gastric residue has lost its virtue of detecting gastrointestinal intolerance to enteral feeding. Thus, in a prospective, randomized study, there was no significant difference in reaching caloric goals or the incidence of ventilator-associated pneumonia between patients where gastric residues were measured or not measured.^(17)^ However, this measurement should be maintained for the assessment of the gastrointestinal tract since this finding together with others, like constipation is associated with an increase in mortality.^(18)^

## Conclusions

We have suggested a pragmatic approach to nutritional support for critically ill patients in the ICU which takes into account their complexity yet provides a uniform, simple approach which can be readily applied. The bundle includes providing nutrition for all patients staying > 2 days in the ICU (in the absence of clear contraindications), initiating enteral feeding early in the ICU course, defining calorie and protein targets and monitoring its achievement. Finally, the appropriate use of parenteral nutrition in the presence of gastrointestinal failure should be positively considered.
